# Endothelial cell-specific molecule 1 (ESM1) promoted by transcription factor SPI1 acts as an oncogene to modulate the malignant phenotype of endometrial cancer

**DOI:** 10.1515/med-2022-0529

**Published:** 2022-08-26

**Authors:** Yu He, Lu Lin, Yurong Ou, Xiaowen Hu, Chi Xu, Caizhi Wang

**Affiliations:** Department of Obstetrics and Gynecology, The First Affiliated Hospital of Bengbu Medical College, Bengbu City, Anhui Province, 233004, China; Department of Obstetrics and Gynecology, The First Affiliated Hospital of Bengbu Medical College, No. 287, Changhuai Road, Bengbu City, Anhui Province, 233004, China

**Keywords:** endometrial cancer, endothelial cell-specific molecule 1, SPI1, proliferation, invasion, malignant phenotype

## Abstract

We aimed to study the function and mechanism of endothelial cell-specific molecule 1 (ESM1) in endometrial cancer (EC). The binding relationship between SPI1 and ESM1 was predicted by bioinformatics analysis and verified by the dual-luciferase reporter assay. The expressions and effects of SPI1 and ESM1 were determined using quantitative real-time PCR, immunohistochemistry, Western blot, and functional experiments. ESM1 was highly expressed in EC and was associated with the poor prognosis of patients. ESM1 silencing suppressed the viability, proliferation, invasion, and angiogenesis of EC cells, down-regulated expressions of PCNA, N-cadherin, Vimentin, VEGFR-1, VEGFR2, and EGFR, but upregulated E-cadherin level, while ESM1 overexpression did oppositely. Moreover, SPI1 bound to ESM1. Overexpressed SPI1 promoted the expression of ESM1 and induced malignant phenotype (viability, proliferation, and invasion), which were countervailed by ESM1 silencing. Collectively, ESM1 induced by SPI1 promotes the malignant phenotype of EC.

## 1 Introduction

Endometrial cancer (EC) refers to the malignant transformation of cells derived from the endometrium [1], which is one of the most common gynecological tumors in the world, causing about 76,000 deaths of women each year [2]. With the changes in people’s life patterns and the adjustment of dietary structure, the incidence of EC is increasing year by year, and the prognosis of EC patients is still poor due to the recurrence and distant metastasis [3]. A recent study on identifying biomarkers to predict the recurrence of the disease and the targets of drug treatment has revealed important insights into the molecular mechanism of EC [4]. Therefore, the further study on EC-related genes is still an important topic for understanding the molecular mechanisms of EC metastasis, invasion, and other malignant biological behaviors.

From the perspective of molecular biology, the pathogenesis of EC includes the gradual change of gene inactivation, tumor suppressor gene mutation, and oncogene activation [5,6]. CDK4 is highly expressed in EC and may be an oncogene, which is closely related to the occurrence and development of EC [7]. Abnormal CpG methylation in the promoter region of RASSF1A tumor suppressor gene leads to EC carcinogenesis, which may affect the invasiveness of tumor [8]. Vascular endothelial growth factor (VEGF) is a key factor involved in angiogenesis, lymphatic vessel formation, and cancer spread and metastasis [9]. Exact studies have shown that VEGF level increases with the progression of EC and an increase in the risk of death [9,10,11]. The above studies have proved that the disorder of the gene level is crucial to the progress of EC. Herein, in-depth exploration of the differentially expressed genes in EC may provide a certain basis for the treatment and prognosis of EC patients.

Endothelial cell-specific molecule 1 (ESM1), a proteoglycan secreted by endothelial cells after activation, has been shown to be widely involved in the growth and metastasis of many tumors, including gastric cancer, kidney cancer, and liver cancer [12,13,14]. For instance, ESM1 expression is apparently upregulated in human head and neck squamous cell carcinoma and may interact with AP-1 [15]. Also, ESM1 has been shown to be highly expressed in EC, but its specific functions are not clear [16]. Combined with the results of our bioinformatics, it is found that the high expression of ESM1 is markedly related to the poor prognosis of EC patients. In addition, the role of transcription factors in EC has received more and more attention [17,18]. To further study the mechanism of ESM1, we used bioinformatics methods to predict the transcription factors that regulate ESM1, and SPI1 has the highest reliability. In light of this, this study mainly takes SPI1/ESM1 as the research object to explore the effect of its abnormal expression on the biological behaviors of EC.

## 2 Materials and methods

### 2.1 Ethics statement

Samples including tumor tissues and the matched adjacent tissues (*n* = 64) were acquired from EC patients who diagnosed in The First Affiliated Hospital of Bengbu Medical College. This research was granted by the Ethics Committee of The First Affiliated Hospital of Bengbu Medical College (XCE202005011). The written informed consents were obtained from all the recruited subjects. Clinicopathological characteristics of EC patients were collected.

### 2.2 Cell culture

Normal endometrial cell lines (EMCs) (CP-H058) and the matching medium (CM-H058) were purchased from Procell Life Science & Technology Co., Ltd. (Wuhan, China). EC cells HEC-1B (HTB-113), HEC-1A (CRL-2692), AN3CA (HTB-111), and RL95-2 (CRL-1671), as well as cell culture medium and supplement including DMEM: F-12 medium (30-2006) and fetal bovine serum (FBS) (30-2020), were provided by American Type Culture Collection (ATCC, USA). Cells were cultivated in an incubator (51033546; Thermo Fisher Scientific, USA) at 37°C with 5% CO_2_ under a humidified atmosphere.

### 2.3 Transfection

The coding sequence of ESM1 or SPI1 was cloned into pcDNA 3.1 vector to obtain the ESM1 and SPI1 overexpression plasmids. shRNAs (ShESM1-1, ShESM1-2) and shNC (shRNA-targeted negative control, C01001) were synthesized from GenePharma (China). The empty vector was used as the negative control (NC). Initially, AN3CA or RL95-2 cells were cultured till 90% confluence was reached. After that, the AN3CA or RL95-2 cells were co-transfected with the above plasmid or vectors using Lipofectamine 3000 reagent (L3000075; Invitrogen, USA) according to the manufacturer’s instructions.

### 2.4 Bioinformatics analysis

GEPIA 2 (http://gepia2.cancer-pku.cn/#index), together with TCGA-UCEC, was used to analyze the expression of ESM1 in EC. The binding sites of ESM1 and SPI1 were analyzed by JASPAR (http://jaspar.genereg.net/analysis) (predicted sequence: GAGAGGAAGGAAGAGAGGGT).

### 2.5 Dual-luciferase reporter assay

Dual-luciferase reporter assay was used to verify the binding of ESM1 and SPI1. Briefly, mutant-type ESM1 sequences were generated by using the QuikChange Multi Site-Directed Mutagenesis kit (Agilent Technologies, Inc.) according to the manufacturer’s protocol. Then, the ESM1-WT (wild-type ESM1 sequences) or ESM1-MUT (mutant-type ESM1 sequences) was cloned into the pmirGLO vector (E1330; Promega, USA) to generate pmirGLO-ESM1-WT and pmirGLO-ESM1-MUT dual-luciferase plasmids. The 293T cells (CRL-3216, ATCC, USA) were co-transfected with recombinant reporter ESM1-WT or ESM1-MUT plasmids and SPI1 overexpression plasmid or NC via Lipofectamine 3000 reagent and cultured for 48 h. Afterwards, relative luciferase activities were determined using dual-luciferase reporter assay kit (E1910; Promega).

### 2.6 Quantitative real-time polymerase chain reaction (qRT-PCR)

Total RNAs were obtained via Triquick reagent (R1100; Solarbio, China), whose quantities and purities were detected utilizing NanoDrop Lite UV-Vis Spectrophotometer (ND-LITE; Thermo Fisher Scientific). Then, the RNAs were reversely transcribed into cDNA using the cDNA synthesis kit (11117831001; Roche, Switzerland). Thereafter, qRT-PCR amplification was carried out in ABI7500 instrument (Applied Biosystems, USA) with the help of universal RT-PCR kit (RP1100; Solarbio). The levels of ESM1, proliferating cell nuclear antigen (PCNA), E-cadherin, N-cadherin, and Vimentin were normalized to that of GAPDH. Primer sequences were listed as below (5′–3′). ESM1: TGGTGAAGAGTTTGGTATCTGC, TTTTCCCGTCCCCCTGTCA; SPI1: GTGCCCTATGACACGGATCTA, AGTCCCAGTAATGGTCGCTAT; PCNA: CCTGCTGGGATATTAGCTCCA, CAGCGGTAGGTGTCGAAGC; E-cadherin: CGAGAGCTACACGTTCACGG, GGGTGTCGAGGGAAAAATAGG; N-cadherin: TCAGGCGTCTGTAGAGGCTT, ATGCACATCCTTCGATAAGACTG; Vimentin: GACGCCATCAACACCGAGTT, CTTTGTCGTTGGTTAGCTGGT; and GAPDH: GGAGCGAGATCCCTCCAAAAT, GGCTGTTGTCATACTTCTCATGG.

### 2.7 Immunohistochemistry (IHC)

The level of ESM1 in EC tissues and adjacent tissues was also detected by IHC. After conventional dewaxing and antigen retrieval, the paraffin tissue sections were then blocked by immunostaining blocking solution (P0102; Beyotime, China) at 4°C overnight. Subsequently, anti-ESM1 antibody (ab224591, dilution ratio 1:100; Abcam, UK) was added to incubate the sections at room temperature for 1 h. Later, the sections were further incubated with horseradish peroxidase-conjugated secondary antibody Goat Anti-Rabbit IgG (ab205718; Abcam) at 37°C for 30 min. Afterwards, the sections were added with DAB chromogenic substrate (DA1015; Solarbio). Finally, the results were observed under a microscope (DMi8; Leica, Germany).

### 2.8 Cell viability assay

EC cell viability was assessed by thiazolyl blue tetrazolium bromide (MTT) kit (M1020), which was purchased from Solarbio. After transfection, the cells (1 × 10^4^ cells) were dissolved with 0.25% trypsin (C0201; Beyotime) and then inoculated into 96-well plates. Twenty-four, forty-eight, or seventy-two hours later, AN3CA or RL95-2 cells were mixed with 90 μL of fresh medium and 10 μL of MTT reagent for another 4-h incubation. After that, 110 μL of formazan dissolving solution was added to each well, subsequent to which the plate was placed on a shaker to shake at low speed for 10 min. At the end, EC cell viability was evaluated by HBS-1096C microplate reader (E0229; Beyotime) at the wavelength of 490 nm.

### 2.9 Cell proliferation assay

Colony formation and anchorage-independent growth assays were performed to determine the proliferation ability of EC cells. For colony formation assay, AN3CA or RL95-2 cells (1 × 10^3^) were cultivated into six-well plates for about 2 weeks, followed by being fixed by methanol and stained with 1% crystal violet (V5265; Sigma-Aldrich, USA). Next, the number of colonies formed was calculated.

For anchorage-independent growth assay, methylcellulose cell clone kit (HL10003) purchased from Shanghai Haring Biological Technology Co., Ltd. (China) was employed. In brief, AN3CA or RL95-2 cells (1 × 10^4^) were seeded in 0.33% basal medium Eagle agar containing 10% FBS and cultured at 37°C with 5% CO_2_. Then, the colonies were calculated after 7 days, and the medium was changed twice a week.

### 2.10 Transwell assay

Transwell invasion assay was performed utilizing the 24-well Transwell chambers (CLS3399; Corning, USA) pre-coated with Matrigel (E1270; Sigma-Aldrich). Shortly, AN3CA or RL95-2 cells (1 × 10^5^) cultured in serum-free medium were put into the upper chamber, while the lower chamber was supplied with the medium containing 10% FBS. After 48 h, the cells were fixed and then stained with 1% crystal violet for 30 min. Finally, cells were counted under a microscope (magnification: ×250).

### 2.11 Tube formation assay

The 24-well plate was pre-coated with Matrigel at 37°C for 60 min. Then, AN3CA or RL95-2 cells (5 × 10^4^) cultured in the medium were inoculated in the plates and incubated for 6 h. After that, the tube formation was observed under a microscope (magnification: ×100).

### 2.12 Western blot

Total proteins were obtained from EC cells through radio-immunoprecipitation assay buffer (R0010; Solarbio). Next, the protein concentration was quantitated by the BCA kit (PC0020; Solarbio). Then, the proteins were separated by 10% sodium dodecyl sulfate–polyacrylamide gel electrophoresis and then transferred onto membranes (2215; Millipore, USA). After being blocked with 5% non-fat milk, membranes were reacted with primary antibodies against PCNA (ab92552, 29 kDa, ½,000), E-cadherin (ab231303, 97 kDa, 1 µg/mL), N-cadherin (ab18203, 100 kDa, 1 µg/mL), Vimentin (ab20346, 54 kDa, 1/1,000), VEGFR-1 (ab32152, 151 kDa, 1/2,000), VEGFR2 (ab11939, 151 kDa, 2 µg/mL), epidermal growth factor receptor (EGFR) (ab52894, 175 kDa, 1/2,000), and GAPDH (ab181602, 36 kDa, 1/10,000). Subsequently, the membranes were incubated with the appropriate secondary antibodies goat anti-rabbit IgG (ab205718, 1/5,000) as well as goat anti-mouse IgG (ab205719, 1/5,000), followed by being exposed to the Enhanced Chemiluminescence Substrate (PE0010; Solarbio) for visualization. All the antibodies were bought from Abcam (USA).

### 2.13 Statistical analysis

All statistical analyses were implemented using Graphpad 8.0 software (Graphpad Prism, USA). Each measurement was performed three times at minimum, the data of which were described as mean ± standard deviation. Paired-sample *t*-test, and one-way analysis of variance were carried out for comparison, followed by Tukey *post hoc* test. Pearson test was conducted to analyze the correlation. Furthermore, Chi-square test or Fisher’s exact probability test was used for count data, and the difference was statistically significant with *P* < 0.05.

## 3 Results

### 3.1 Increased expression of ESM1 was related to the prognosis of EC patients

**Figure 1:**
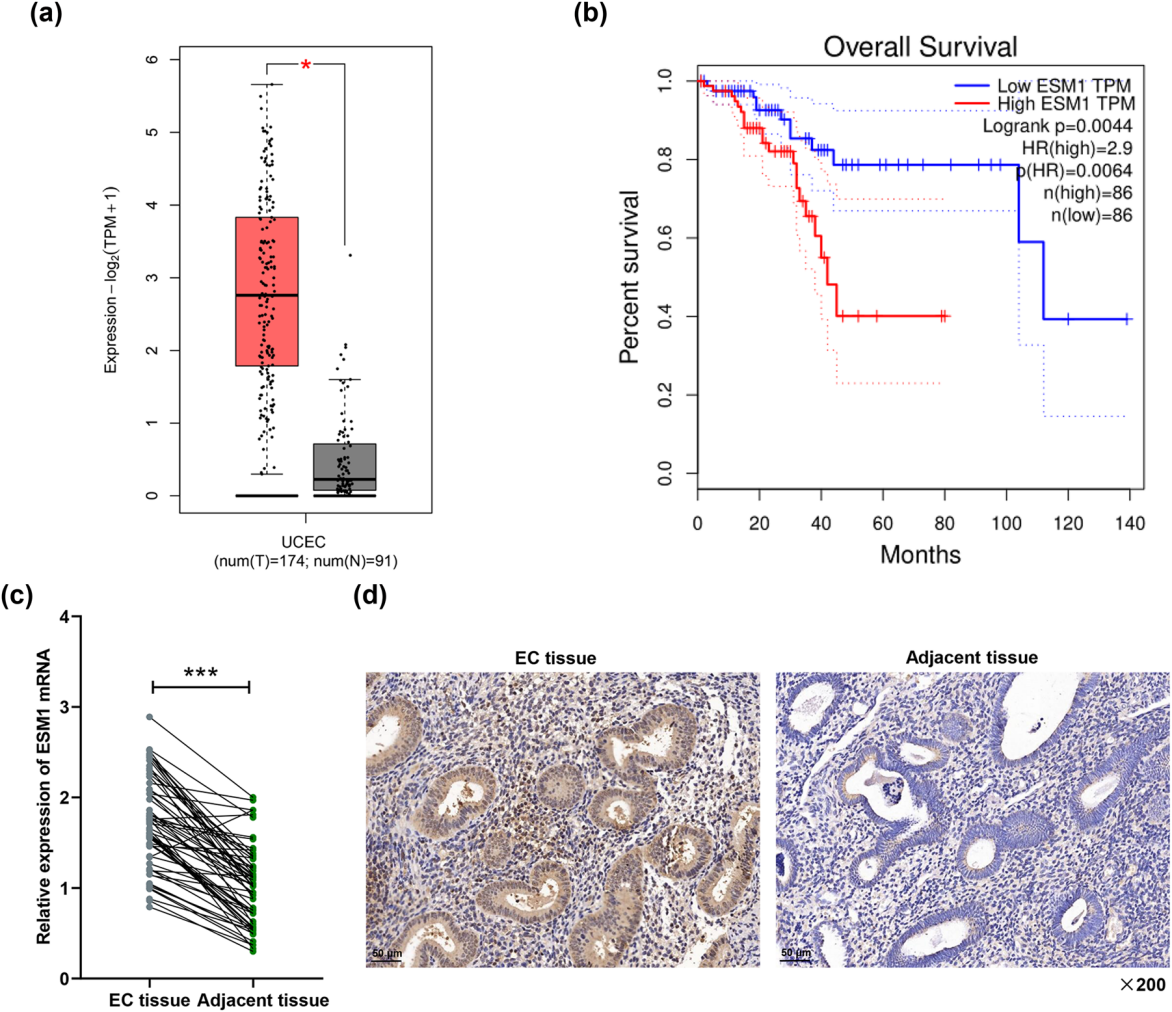
ESM1, which was highly expressed in EC, and patients with high expression of ESM1 had a poor prognosis. (a) The expression of ESM1 in UCEC was analyzed, and ESM1 was highly expressed in EC. (b) High ESM1 expression was significantly associated with the poor prognosis of EC patients. (c) The cancer tissues and adjacent tissues of 64 EC patients were collected to determine the expression of ESM1 by qRT-PCR. (d) The expression of ESM1 in the cancer tissues and adjacent tissues of EC patients was detected by immunohistochemical staining (magnification: ×200 times). GAPDH was used as a standardized gene. The experimental data are represented as mean ± standard deviation. ^***^ *P* < 0.001 vs adjacent tissue.

Through TCGA-UCEC analysis, we found that ESM1 expression was upregulated in EC, and it was significantly related to the poor prognosis of patients (*P* < 0.05, Figure 1a and b). The same result on the expression of ESM1 in clinical samples was obtained by qRT-PCR and immunohistochemical staining assay. Compared with adjacent tissues, ESM1 expression was significantly higher in EC tissues (*P* < 0.001, Figure 1c and d). Moreover, elevated ESM1 expression was significantly associated with higher histology grade, deeper depth of infiltration, lymph node metastasis, and TNM stage (*P* < 0.05, Table 1), suggesting that ESM1 may be a potential marker for clinical prognosis.

**Table 1:** Relationship between the expression of ESM1 and the clinical characteristics of endometrial cancer patients

Clinicopathlogical features	Cases (*n*)	ESM1 expression		*P*-value
		High (*n* = 32)	Low (*n* = 32)	
Menopause				
Yes	35	18	17	0.801
No	29	14	15	
Age (years old)				
<50	45	20	25	0.171
≥50	19	12	7	
Histology grade				
Ⅰ	27	8	19	0.025
Ⅱ	16	10	6	
Ⅲ	21	14	7	
Depth of infiltration				
Shallow	24	8	16	0.039
Deep	40	24	16	
Lymph node metastasis				
Yes	29	19	10	0.024
No	35	13	22	
TNM stage				
Ⅰ–Ⅱ	28	8	20	0.003
Ⅲ–Ⅳ	36	24	12	

### 3.2 ESM1 overexpression promoted the viability and proliferation of EC cells

**Figure 2:**
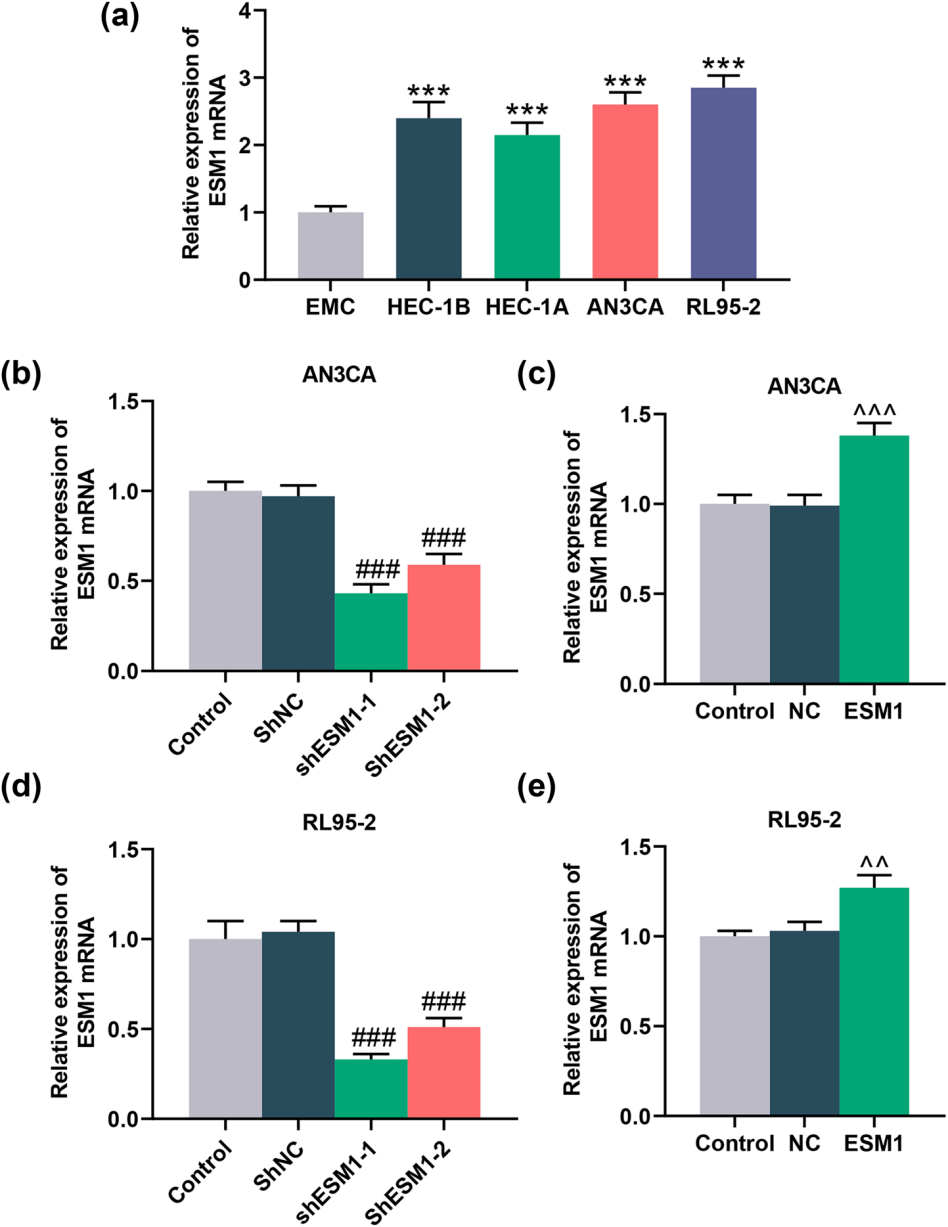
Expression of ESM1 in EC cells. (a) qRT-PCR was applied to quantify the expression of ESM1 in EMCs and EC cells (HEC-1B, HEC-1A, AN3CA, and RL95-2). (b and c) After AN3CA cells were transfected with shESM1-1, shESM1-2, or ESM1 overexpression plasmid, the level of ESM1 in AN3CA cells was determined by qRT-PCR. (d and e) After RL95-2 cells were transfected with shESM1-1, shESM1-2, or ESM1 overexpression plasmid, the level of ESM1 in RL95-2 cells was determined by qRT-PCR. GAPDH was applied as a standardized gene. ^***^ *P* < 0.001 vs EMC; ^###^ *P* < 0.001 vs short hairpin RNA-targeted negative control (shNC); ^^^^ *P* < 0.01, ^^^^^ *P* < 0.001 vs NC.

To further delve into the possible role of ESM1 in EC, we conducted cell experiments as appropriate. As shown in Figure 2a, the expression of ESM1 in EC cells (HEC-1B, HEC-1A, AN3CA, and RL95-2) was greatly increased compared with that in EMCs (*P* < 0.001). Among these EC cells, AN3CA and RL95-2 cells exhibited relatively higher ESM1 expression, which were thereby selected for the subsequent studies. After the transfection of AN3CA and RL95-2 cells with shESM1-1, shESM1-2 and ESM1 overexpression plasmid, we found that shESM1-1 and shESM1-2 successfully reduced the expression of ESM1, while the ESM1 overexpression plasmid obviously increased the expression of ESM1 (*P* < 0.01, Figure 2b–e). Since shESM1-1 had a more pronounced silencing effect, it was used in subsequent studies.

**Figure 3:**
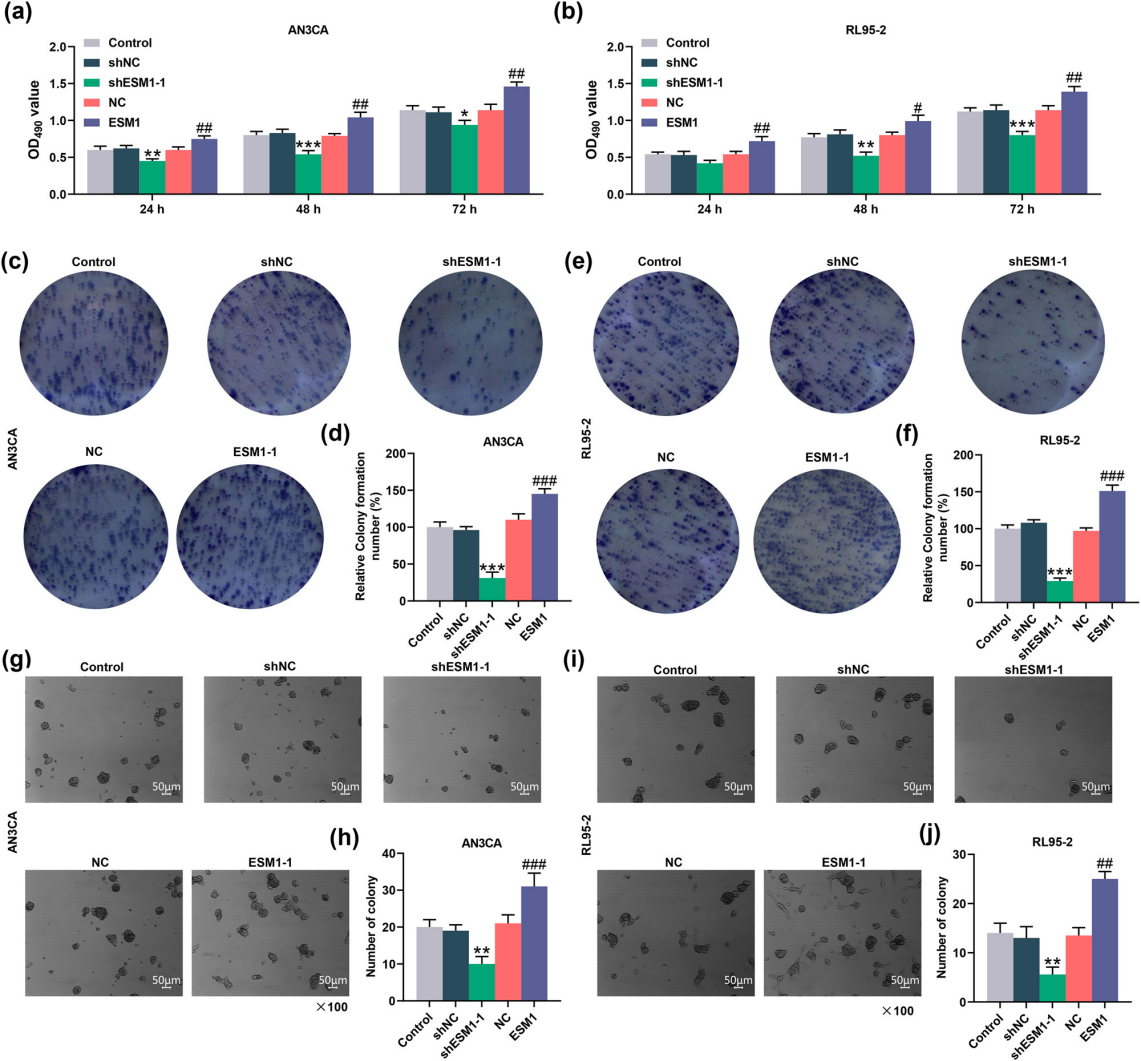
ESM1 overexpression promoted the viability and proliferation of EC cells, while silencing of ESM1 had opposite effects. (a and b) 24, 48, and 72 h after cell transfection with shESM1-1 or ESM1 overexpression plasmids, ESM1 overexpression markedly promoted the viability of AN3CA and RL95-2 cells, while sh-ESM1 worked conversely, as measured by MTT assay. (c–f) Colony formation assay was employed to evaluate the effect of ESM1 silencing or its overexpression on the colony-forming abilities of AN3CA and RL95-2 cells. The representative image was presented and relative colony formation was detected. (g–j) After transfection with silencing or overexpression plasmid of ESM1 in AN3CA and RL95-2 cells, cell anchorage-independent assay was conducted to detect the cell anchorage-independent proliferation potential. ^*^ *P* < 0.05, ^**^ *P* < 0.01, ^***^ *P* < 0.001 vs short hairpin RNA-targeted negative control (shNC); ^#^ *P* < 0.05, ^##^ *P* < 0.01, ^###^ *P* < 0.001 vs NC.

Next, we explored the effects of ESM1 on the viability and proliferation of AN3CA and RL95-2 cells. The result showed that ESM1-1 silencing obviously inhibited the viability of AN3CA and RL95-2 cells at 24, 48, and 72 h, while ESM1 overexpression promoted the viability of AN3CA and RL95-2 cells at 24, 48, and 72 h (*P* < 0.05, Figure 3a and b). In addition, silencing of ESM1 decreased the colony formation of AN3CA and RL95-2 cells, whereas ESM1 overexpression ran inversely (*P* < 0.001, Figure 3c–f). Moreover, cell anchorage-independent assay also confirmed that silencing of ESM1 impeded the proliferation, while ESM1 overexpression augmented the proliferation of AN3CA and RL95-2 cells (*P* < 0.01, Figure 3g–j).

### 3.3 ESM1 overexpression enhanced the invasion and angiogenesis of EC cells

**Figure 4:**
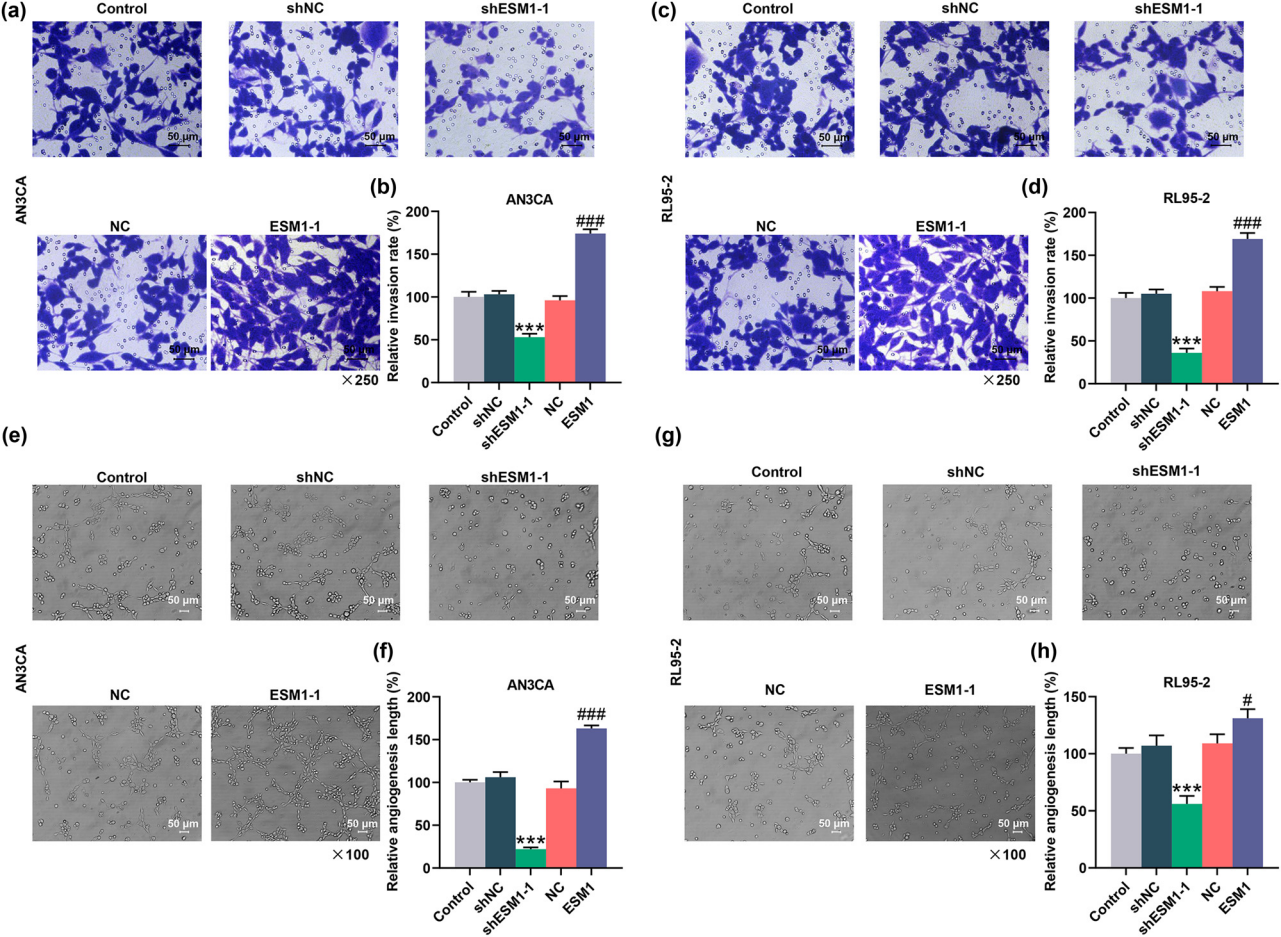
ESM1 overexpression promoted EC cell invasion and angiogenesis, while silencing of ESM1 had opposite effects. (a–d) The invasion of AN3CA or RL95-2 cells in the control, shNC, short hairpin RNA-targeted ESM1 (shESM1-1), NC, and ESM1 groups was detected by Transwell assay. The representative image was presented (magnification: ×250 times; scale bar: 50 µm) and relative invasion rate was quantified. (e–h) Tube formation assay was performed to assess the effect of ESM1 overexpression or silencing on angiogenesis. The representative image was presented (magnification: ×100 times; scale bar: 50 µm) and angiogenesis length was measured. ^***^ *P* < 0.001 vs Sh-NC; ^#^ *P* < 0.05, ^###^ *P* < 0.001 vs NC.

The effects of ESM1 on the invasion and angiogenesis of AN3CA and RL95-2 cells were detected. The invasion was found to be inhibited after AN3CA and RL95-2 cells were transfected with shESM1-1. However, the invasion of AN3CA and RL95-2 cells was promoted following transfection of ESM1-1 overexpression plasmid (*P* < 0.001, Figure 4a–d). In addition, compared with the shNC group, the angiogenesis length was shorter in the shESM1-1 group. Moreover, the angiogenesis length was longer in the ESM1 group than that in the NC group (*P* < 0.001, Figure 4e–h). These results indicated that ESM1 overexpression promoted the invasion and angiogenesis of AN3CA and RL95-2 cells.

### 3.4 ESM1 affected genes related to proliferation, epithelial-mesenchymal transition (EMT), and angiogenesis in EC cells

**Figure 5:**
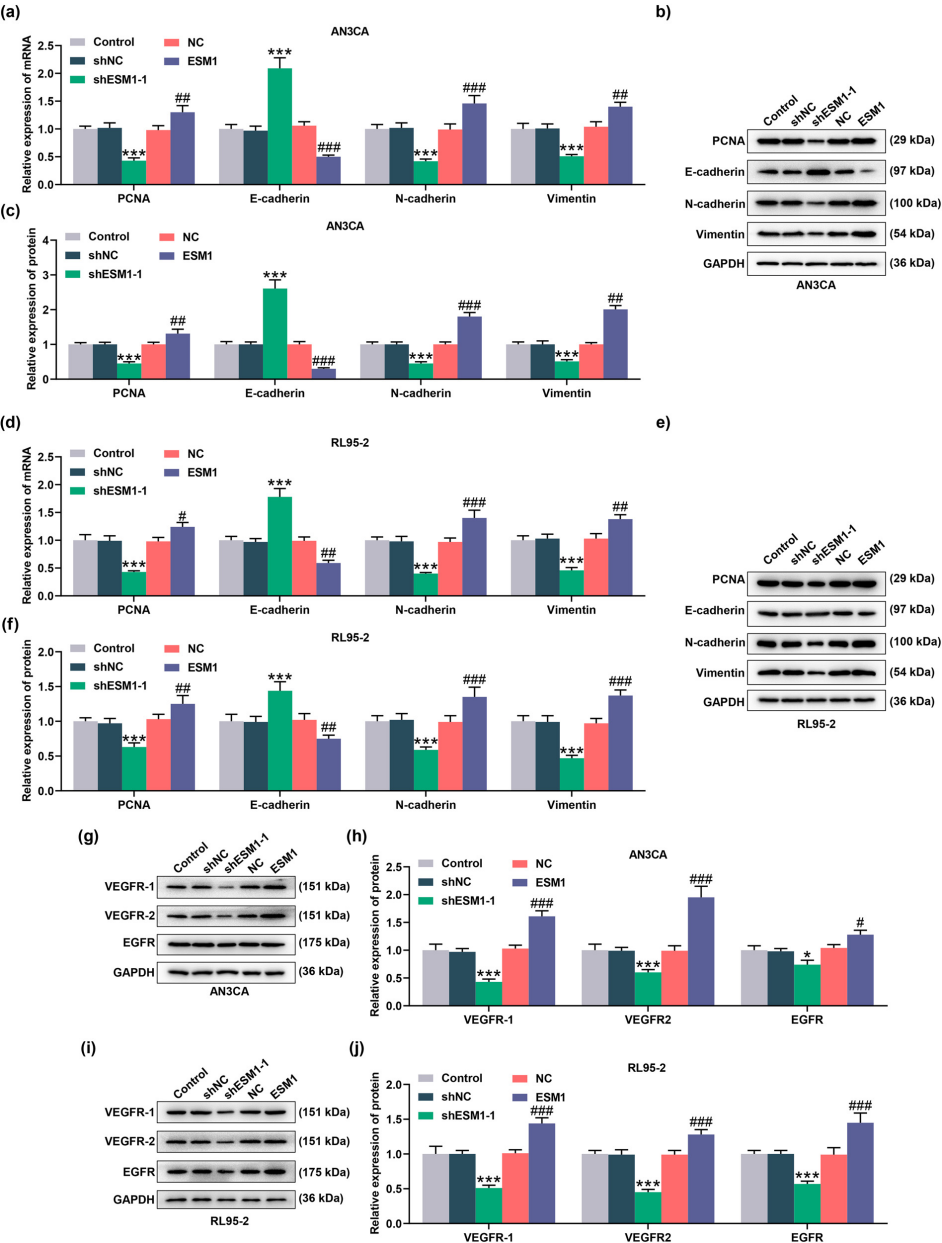
ESM1 affected the proliferation-, epithelial-mesenchymal transition (EMT)-, and angiogenesis-related proteins in EC cells. (a–f) The effects of ESM1 overexpression or its silencing on the mRNA and protein expressions of PCNA, E-cadherin, N-cadherin, and Vimentin were evaluated by qRT-PCR and Western blot. (g–j) Western blot was used to detect the protein expressions of VEGFR1, VEGFR2, and epidermal growth factor receptor (EGFR) in AN3CA or RL95-2 cells. GAPDH was selected as the internal reference gene. ^*^ *P* < 0.05, ^***^ *P* < 0.001 vs ShNC; ^#^ *P* < 0.05, ^##^ *P* < 0.01, ^###^ *P* < 0.001 vs NC.

To better explore the action mechanism of ESM1 in EC, the levels of mRNA and proteins related to proliferation, EMT, and angiogenesis were measured (*P* < 0.05, Figure 5a–j). It turned out that silencing of ESM1 reduced the expression levels of PCNA, N-cadherin, Vimentin, VEGFR-1, VEGFR2, and EGFR but increased the expression of E-cadherin in AN3CA and RL95-2 cells (*P* < 0.05). On the contrary, the overexpression of ESM1 effectively promoted the expression of PCNA, N-cadherin, Vimentin, VEGFR-1, VEGFR2, and EGFR, while significantly suppressing the expression of E-cadherin in AN3CA and RL95-2 cells (*P* < 0.05).

### 3.5 ESM1 was induced by SPI1, which was highly expressed in EC

**Figure 6:**
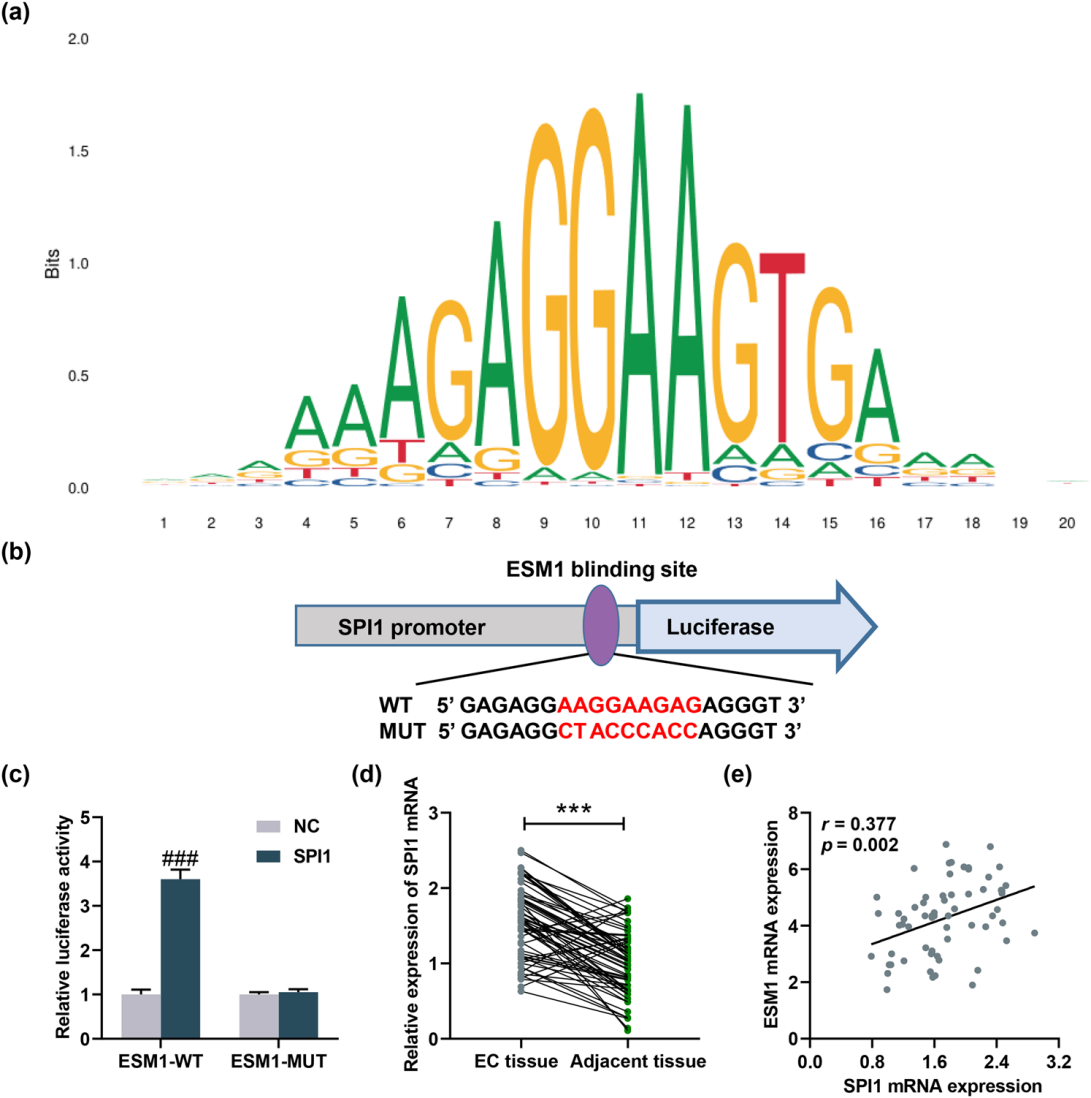
ESM1 was induced by SPI1, which was highly expressed in EC. (a) The motif logo of SPI1 was analyzed by JASPAR (http://jaspar.genereg.net/analysis). (b) The ESM1-binding sites with the SPI1 promoters. (c) SPI1 bound to ESM1, which was verified by dual-luciferase reporter assay. (d) The expression of SPI1 in the cancer tissues and adjacent tissues of 64 EC patients was quantified by qRT-PCR, and SPI1 was highly expressed in EC tissues. (e) The correlation between SPI1 expression and ESM1 in EC tissues was analyzed by *Pearson* test, the results of which indicated that SPI1 was positively related to ESM1. ^***^ *P* < 0.001 vs EC tissue; ^###^ *P* < 0.001 vs NC.

**Figure 7:**
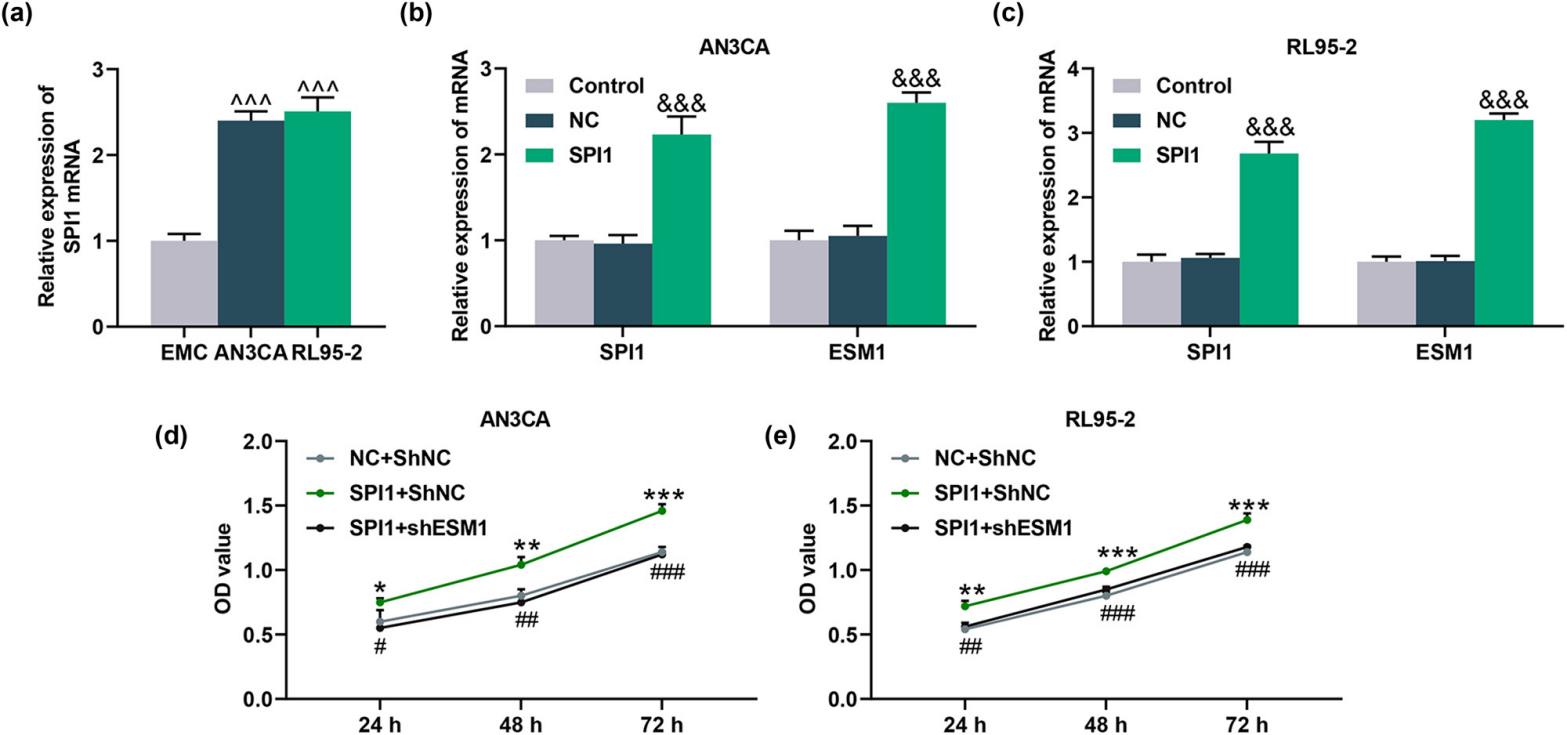
SPI1 overexpression promoted ESM1 level and viability of EC cells, and silencing of ESM1 reversed the effect of SPI1 overexpression on the viability of EC cells. (a) The expression of SPI1 in EMCs as well as AN3CA and RL95-2 cells was detected by qRT-PCR, and SPI1 was highly expressed in AN3CA and RL95-2 cells. (b and c) After AN3CA and RL95-2 cells were transfected with SPI1 overexpression plasmid, the expression levels of SPI1 and ESM1 were quantified by qRT-PCR. (d and e) Twenty-four, forty-eight, or seventy-two hours after cell transfection with SPI1 overexpression plasmid and shESM1, the effects of overexpressed SPI1 and silent ESM1 on the viability of AN3CA and RL95-2 cells were evaluated by MTT assay. ^^^^^ *P* < 0.001 vs EMC; ^&&&^ *P* < 0.001 vs NC; ^*^ *P* < 0.05, ^**^ *P* < 0.01, ^***^ *P* < 0.001 vs NC + ShNC; ^#^ *P* < 0.05, ^##^ *P* < 0.01, ^###^ *P* < 0.001 vs SPI1 + ShNC.

According to the prediction of bioinformatics analysis, we discovered that the transcription factor SPI1 bound to ESM1, which was verified by dual-luciferase reporter assay. The motif logo of SPI1 is shown in Figure 6a, and the binding site is shown in Figure 6b. When compared with 293T cells co-transfected with NC and ESM1-WT vector, the luciferase activity was increased in 293T cells co-transfected with SPI1 overexpression plasmid and ESM1-WT vector, with no change in the luciferase activity observed in 293T cells co-transfected with NC or SPI1 overexpression plasmid and ESM1-MUT vector (*P* < 0.001, Figure 6c). Afterwards, we found that SPI1 was highly expressed in EC tissues compared with that in adjacent tissues (*P* < 0.001, Figure 6d). In addition, we analyzed the correlation between ESM1 and SPI1 in EC tissues and discovered that SPI1 expression was positively correlated with ESM1 expression (*r* = 0.377, *P* = 0.002, Figure 6e). Similarly, SPI1 was highly expressed in AN3CA and RL95-2 cells (*P* < 0.001, Figure 7a).

### 3.6 ESM1 silencing reversed the promotive effect of SPI1 overexpression on the viability, proliferation, and invasion of EC cells

**Figure 8:**
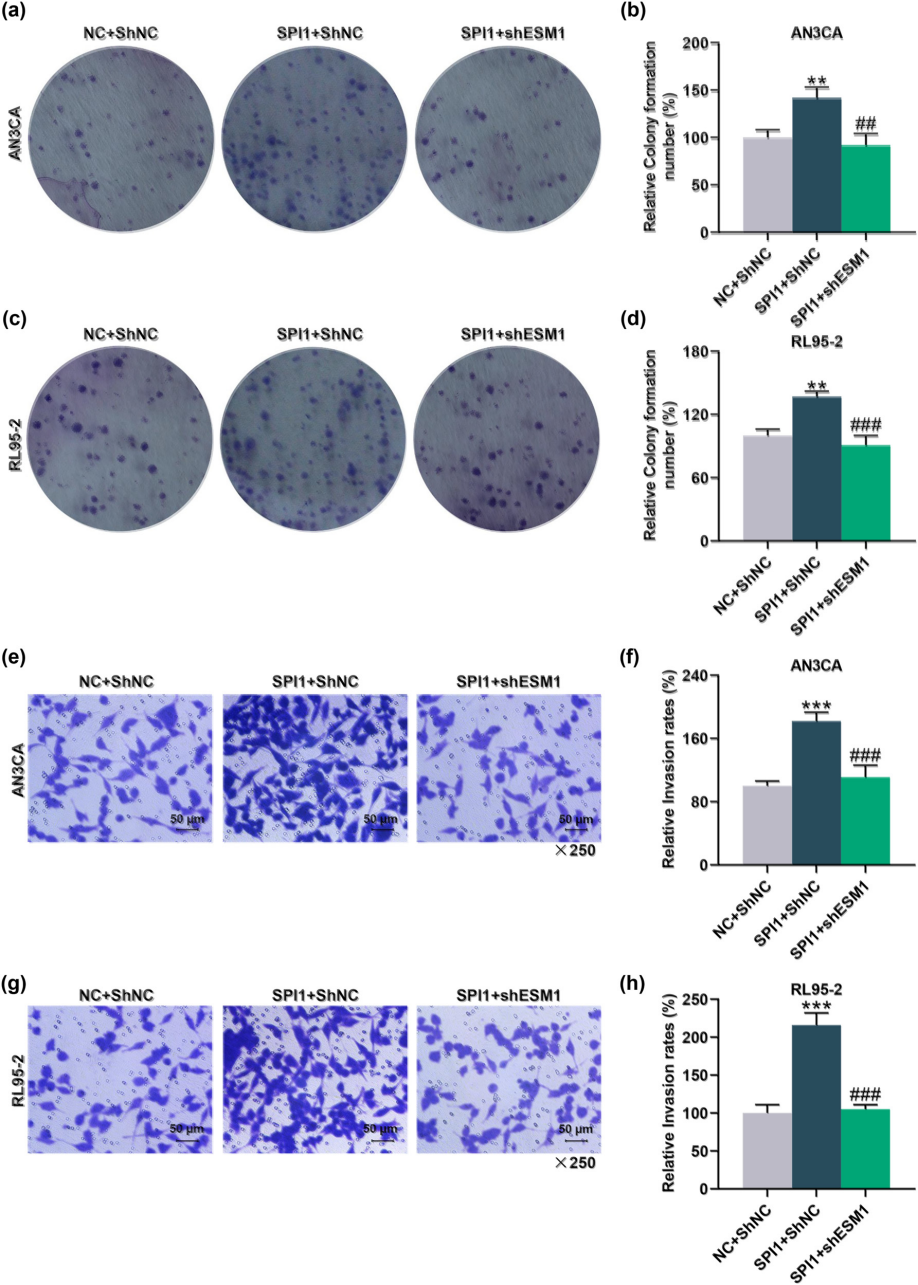
Silencing of ESM1 reversed the promotive effects of SPI1 overexpression on the proliferation and invasion of EC cells. (a–d) Colony formation assay was applied to detect the effects of SPI1 overexpression and ESM1 silencing on the colony-forming abilities of AN3CA and RL95-2 cells. (e–h) The invasion of AN3CA or RL95-2 cells in each group was detected by Transwell assay (magnification: ×250 times; scale bar: 50 µm). ^**^ *P* < 0.01, ^***^ *P* < 0.001 vs NC + ShNC; ^##^ *P* < 0.01, ^###^ *P* < 0.001 vs SPI1 + ShNC.

Overexpressed SPI1 markedly enhanced SPI1 and ESM1 levels in AN3CA and RL95-2 cells (*P* < 0.001, Figure 7b and c). Function experiments showed that overexpressed SPI1 increased the OD value of AN3CA and RL95-2 cells at 24, 48, and 72 h, while silencing of ESM1 reversed the promotive effect of SPI1 overexpression on the OD value of AN3CA and RL95-2 cells (*P* < 0.05, Figure 7d and e). In addition, the effect of SPI1 overexpression on the proliferation and invasion was also detected by colony formation and Transwell assays. The result showed that the colony formation number and the invasion rate were increased after AN3CA and RL95-2 cells were transfected with SPI1 overexpression plasmid, while silencing of ESM1 reversed the effects of SPI1 overexpression on the colony-forming ability and the invasion rate (*P* < 0.001, Figure 8a–h). Taken together, ESM1 silencing offset the promotive effect of SPI1 overexpression on the viability, proliferation, and invasion of EC cells.

## 4 Discussion

Understanding the pathogenesis of EC at the molecular level is of great help to its prevention and treatment [1]. The continuous discovery and research of tumor markers provide an important research direction for the further diagnosis and treatment of EC [1,2]. This study demonstrated that ESM1 is highly expressed in EC and is related to poor prognosis. In addition, at the cellular level, we proved that overexpression of ESM1 promotes the malignant biological phenotype of EC. On the contrary, silencing of ESM1 inhibits the malignant transformation of EC cells, suggesting that ESM1 may be a potential biomarker for the diagnosis, treatment, and prognosis of EC. Importantly, SPI1 can induce the upregulation of ESM1 in EC cells and regulate the effect of ESM1 on EC cell functions, partially filling the gap in the current mechanism of ESM1 in EC.

ESM1 was first discovered as a soluble dermatan sulfate proteoglycan secreted by endothelial cells and then gradually reported to play a vital role in a variety of cancers [12,14]. ESM1 is regarded as a new type of tumor marker, which has been shown to be related to cancer cell proliferation and angiogenesis [19], and can act as a target as well as regulator of VEGF in endothelial cells [20]. In the research of breast cancer, ESM1 was found to further enhance the malignant transformation of triple-negative breast cancer cells by activating the AKT/NF-κB/Cyclin D1 pathway [21]. Not only that, Jin et al. revealed the effects of ESM1 and radioresistance and found that the upregulation of ESM-1 is involved in the tumorigenesis of radiation-resistant breast cancer cells [22]. Coincidentally, Feng et al. pointed out that ESM1 expression is enhanced in bladder cancer and has the function of promoting the progression of bladder cancer cells [23]. Nevertheless, ESM1 has rarely been studied in EC, with a report on its abnormal expression only [16]. In addition, we proved the role of ESM1 as an oncogene in EC. The above research results prove that ESM1 is a biomarker worth looking forward to.

To explain the regulation effect of ESM1 on EC, we detected the key protein levels associated with the tumorigenesis and development of EC. A key factor in tumor development is the survival and proliferation of cells with malignant potential [24]. As a typical proliferation marker, PCNA has been affirmed to affect EC cell proliferation [25]. As expected, the upregulation of ESM1 promotes PCNA expression in EC cells. Another major feature of EC cells is that they are prone to epithelial–mesenchymal transition (EMT) [26,27]. E-cadherin, N-cadherin, and Vimentin are classic indicators of the EMT process [28]. Upregulation of N-cadherin and Vimentin as well as downregulation of E-cadherin indicate that tumor cells are more prone to EMT [29]. Here, we proved that ESM1 may promote the EMT process by affecting EMT-related genes. In addition, angiogenesis is a key factor in tumor progression because it provides oxygen and nutrients to the actively proliferating tumor cells [30]. VEGFR1 and VEGFR2 are the key factors of EC angiogenesis [31]. Furthermore, EGFR has been reported to be involved in the development of multiple cancer types, including EC [32,33]. Notably, this study revealed that ESM1 promoted the change of EGFR protein. Therefore, we believe that ESM1 may affect the biological characteristics of EC cells by regulating a series of proteins related to proliferation, EMT, and angiogenesis.

It should also be noted that SPI1 is predicted and verified to promote ESM1 expression in EC cells. SPI1 also frequently appears in cancer. For example, Tao et al. pointed out that SNHG16 promotes the expression of PARP9 by recruiting SPI1, thereby promoting the malignant progression of cervical cancer cells [34]. SPI1 expression is upregulated in glioma and is involved in the progression of glioma [35]. MeCP2 binds to the transcription factor SPI1 and promotes the expression of ZEB1 at the transcription level to promote colorectal cancer metastasis [36]. Here, we found that ESM1 silencing reversed the effect of SPI1 on EC cells, implying that the transcription factor SPI1 activates the expression of ESM1 to accelerate the progression of the malignant phenotype of EC cells.

In summary, our results show that EC cells containing high expression of ESM1 are highly vascularized, with rapid growth and high aggressiveness, involving a poor prognosis of patients. Our study provides evidence that ESM1 promotes the malignant transformation of EC cells *in vitro*. Clinically, abnormal expression of ESM1 is associated with the poor prognosis of patients. Mechanistically, SPI1 binds to the ESM1 promoter and activates ESM1 expression. In combination with the above findings, it is plausible to conclude that ESM1 induced by SPI1 may promote the development of EC. Remarkably, combined application of tumor markers in clinical obstetrics and gynecology may provide effective help for early diagnosis and prognosis of EC patients in clinic. Nevertheless, the regulatory network of SPI1/ESM1 in EC still needs to be further explored.
